# Community-acquired antimicrobial resistance among Syrian refugees and the local population in Türkiye

**DOI:** 10.1093/eurpub/ckad119

**Published:** 2023-07-19

**Authors:** Serap Süzük Yıldız, Can Hüseyin Hekimoğlu, Mustafa Bahadır Sucaklı, Zekiye Bakkaloğlu, Yasemin Numanoğlu Çevik, Özlem Ünaldı, Hayal Arslantürk, Monica Zikusooka, Melda Keçik, Laura Nellums, Omur Cinar Elci

**Affiliations:** Department of Microbiology Reference Laboratory and Biological Product, Ministry of Health, General Directorate of Public Health, Ankara, Türkiye; Department of Microbiology Reference Laboratory and Biological Product, Ministry of Health, General Directorate of Public Health, Ankara, Türkiye; World Health Organization, Türkiye Country Office, Ankara, Türkiye; Department of Microbiology Reference Laboratory and Biological Product, Ministry of Health, General Directorate of Public Health, Ankara, Türkiye; Department of Microbiology Reference Laboratory and Biological Product, Ministry of Health, General Directorate of Public Health, Ankara, Türkiye; Department of Microbiology Reference Laboratory and Biological Product, Ministry of Health, General Directorate of Public Health, Ankara, Türkiye; Department of Microbiology Reference Laboratory and Biological Product, Ministry of Health, General Directorate of Public Health, Ankara, Türkiye; World Health Organization, Türkiye Country Office, Ankara, Türkiye; World Health Organization, Türkiye Country Office, Ankara, Türkiye; Lifespan and Population Health, School of Medicine, University of Nottingham, Nottingham, UK; World Health Organization, Türkiye Country Office, Ankara, Türkiye; Clinical & Behavioral Medicine, Western Atlantic University School of Medicine Freeport, Grand Bahama, The Bahamas

## Abstract

**Background:**

The long-standing antimicrobial resistance (AMR) pandemic has proven difficult to resolve and is becoming more complex, especially in the context of increasing forced migration, with little evidence around patterns of AMR in migrant communities. This study aimed to determine the frequency in the carriage of common types of antimicrobial-resistant bacteria between Syrian refugees and the local communities in Türkiye: extended-spectrum β-lactamase (ESBL), methicillin-resistant *Staphylococcus aureus* (MRSA) and vancomycin-resistant enterococci (VRE).

**Methods:**

We collected nasal swabs and stool samples from the study participants, the local community members, and refugees, between September 2020 and March 2021. We screened clinical samples for the presence of ESBL, MRSA and VRE. Antimicrobial-resistant bacterial isolates were tested by phenotypic analysis to determine the AMR status.

**Results:**

The study included a total of 3960 participants: 1453 individuals in the local community (36.2%) and 2525 Syrian refugees (63.8%). Overall, a significantly greater proportion of refugees (6.7%) carried MRSA compared to the local community (3.2%) (*P* < 0.001). The ESBL-positivity rate was 17.9% in Syrian refugees and 14.3% in the local community (*P* = 0.041). Carbapenemase activity was detected in three isolates from Syrian refugees. No VRE was detected in Syrian refugees or the local community.

**Conclusions:**

This large, community-based study on the frequency and the distribution of AMR among Syrian refugees and the local population is the first study in Türkiye.

## Introduction

Antimicrobial resistance (AMR) is a major global health problem in both hospitals and community settings. Elevated rates of AMR have been documented in low- and middle-income countries, which can be attributed to healthcare disparities, poor public health infrastructure, hygiene and sanitation measures, access to and consumption of antimicrobials and the quality of drugs. Conditions such as large-scale armed conflicts or disasters can further impact these contributing factors. Evidence shows that an increased movement of people, including both travel and migration, has contributed to the spread of AMR globally.^1^ However, it is hypothesized that the migration journey alone could also be one of the determinants of AMR among migrants, rather than countries of origin being solely responsible for the importation of drug-resistant organisms. Evidence suggests that, due to conflict situations, migrants may be at higher risk conditions, or they may be acquiring drug-resistant organisms in transit or host countries. Refugees may show particular vulnerability to AMR exposure during and after migration due to factors such as sanitation, hygiene and overcrowding in refugee camps, transit centers and detention facilities, with limited access to health services in host countries.^2^ However, only limited data exist on the prevalence of AMR carriage or infection among refugees and migrants, particularly for those who have been forcibly displaced. There is a need for robust estimates of the AMR burden in communities receiving a high number of migrants to facilitate the detection, appropriate treatment and prevention of AMR in receiving countries, which may have different AMR profiles.^3^ Although AMR is among the leading causes of death, the burden of AMR is mostly monitored in resource-limited regions, where data are always more difficult to obtain.^4^ Therefore, it is important both to improve data quality to conduct surveillence studies, and to ensure sustainable management to develop prevention strategies for AMR.^5^

Türkiye is a country of origin, transit and destination for migrants since it is a crossroads between Europe and Asia.^3^ During the initial years of the Syrian crisis, between 2011 and 2016, the Turkish government settled Syrian refugees in conventional refugee camps in the provinces bordering Syria. Currently, only 46 079 (1.26%) of the recorded ∼3.7 million Syrian immigrants in Türkiye are settled in camps. As per government policy, the remaining Syrian refugees (over 98%) are settled in residential neighborhoods throughout the country.^3^^,^^6^ In addition to the Syrian conflict, due to other regional conflicts, there are over 4 million recorded immigrants who have been residing in Türkiye.^6^ Due to Türkiye’s geopolitical position as a transit route between Europe, Asia and Africa, robust population estimates of AMR carriage in the community are important to inform health policy and public health decision-making. This is necessary to ensure sustainable management, provide equitable services, and reduce the burden of disease in both migrant and local populations. However, no population-based evidence is available on AMR patterns in Türkiye.

This study aimed to examine the frequency and distribution patterns of AMR carriage of common types of antimicrobial-resistant bacteria in the community, including extended-spectrum β-lactamase (ESBL), methicillin-resistant *Staphylococcus aureus* (MRSA) and vancomycin-resistant enterococci (VRE), among Syrian refugees and the local community members in Türkiye.

## Methods

### Study design

In this cross-sectional population-based study, we selected seven provinces (Ankara, Gaziantep, Hatay, Istanbul, Izmir, Mersin and Sanliurfa) with the largest Syrian refugee populations (*n* = 2 309 074; 63.0% of the total refugee population) in Türkiye as a study population. The estimated sample size for each province was calculated using WinPepi electronic epidemiological calculator, version 11.65. A 95% confidence interval (95% CI) estimated AMR carriage prevalence of 50%, a 0.05 margin of error and an additional 10% loss to follow-up were assumed. Inclusion criteria for all study participants were that participants should be residents of the province and aged 5 years or older and have: no symptoms of infection, have never been treated with chemotherapy and have never been diagnosed with cancer. Study participants were enrolled in Family Health Centers (FHCs), where only Turkish citizens received healthcare services, and in Refugee Health Centers (RHCs) where only Syrian refugees received healthcare services. The overall estimated sample size from both FHCs and RHCs was 6116. Each isolate included in the study was obtained from one participant. The workflow for the study is presented in Supplementary figure S1.

The study faced a major obstacle at the initial data collection stage, due to the spread of the SARS-CoV-2 (COVID-19) pandemic. Data collection became unfeasible in the capital city, Ankara, where the national COVID-19 pandemic management center was established. Therefore, Ankara province was removed from the study, leaving a total of six provinces. Culturally sensitive, informed consent was received from all study participants. The study information was provided in a linguistically and culturally appropriate format, in particular to Syrian refugee participants. For that purpose, we employed healthcare professionals who are themselves refugees. In case of need for translation, bilingual trained patient guides helped the study team to support interpretation and data collection.

### Collection of demographic data and biological samples

After receiving training on the objectives and methodology of the study, obtaining informed consent, sample collection and shipping to the laboratory, a total of 831 healthcare workers in the centers were involved in the study. All data and samples were collected between 1 September 2020 and 31 March 2021.

### Analysis of nasal swab samples

Nasal swabs were incubated on an *S. aureus* selective medium (mannitol salt agar; HiMedia Laboratories, India). All bacterial isolates were identified by MALDI-TOF MS (Bruker MALDI Biotyper, Germany). Confirmed *S. aureus* isolates were assessed for methicillin resistance using the disk diffusion method with 30 µg cefoxitin disks (HiMedia Laboratories, India). Controls were *S. aureus* NCTC 12493, *S. aureus* NCTC 13552 and *S. aureus* ATCC 25923 (negative control).

### Analysis of stool samples

To identify VRE, stool samples were inoculated onto VRE agar (HiCrome VRE Agar, HiMedia Laboratories). To identify Gram-negative bacilli, stool samples were inoculated onto eosin methylene blue agar. All isolates were identified by MALDI-TOF MS (Bruker MALDI Biotyper, Germany). Antibiotic susceptibility was assessed using the disk diffusion method using the following antibiotics: 30 µg cefepime, 5 µg cefotaxime, 10 µg ceftazidime, 30 µg ceftriaxone, 5 µg ciprofloxacin, 10 µg ertapenem, 10 µg gentamicin, 10 µg imipenem, 10 µg meropenem and 36 µg piperacillin–tazobactam (HiMedia Laboratories, India). Suspected isolates were tested for the production of ESBLs with the combination disk test.^7^ Isolates resistant to cefoxitin, and cefotaxime or ceftazidime were tested for the production of AmpC β-lactamases (AmpC) using a disk test (Mast Discs Combi, UK). All disc diffusion tests were evaluated according to the European Committee on Antimicrobial Susceptibility Testing (EUCAST).^7^ Multi-drug resistance (MDR) is defined as combined resistance to third-generation cephalosporins [cefotaxime or ceftazidime or ceftriaxone, fluoroquinolones (ciprofloxacin) and aminoglycosides (gentamycin)]. No VRE was identified.

### Statistical analysis

Data were analyzed with SPSS version 15.0 (SPSS Inc., USA) using descriptive statistics (mean and standard deviation, number and percentages).^8^ Nominal variables were compared using the Chi-square (χ^2^) test and Fisher’s exact test. 95% CIs were calculated using the Score (Wilson) method with OpenEpi version 3.01.^9^ Continuous variables were compared using the independent sample Student *t*-test.

### Ethical consideration

A local ethical review of this study has been provided by Health Sciences University, Ankara City Hospital Ethical Review Committee. Additional institutional approval has been received from the MoH (E-19-007). Furthermore, the WHO Ethical Review Committee has also approved the study (ERC.0003212).

## Results

### Analysis of demographic data

A total of 3960 individuals participated in the study, including 1435 (36.2%) individuals from the local community in Türkiye, and 2525 (63.8%) Syrian refugees. Supplementary table S1 shows the number of participants and the number of biological samples collected from each province. The average age of participants was 35.6 ± 17.1 years for the local community and 30.0 ± 15.0 years for Syrian refugees. Among all, 36.7% of participants claimed they had not used any antibiotics in the last 6 months, and 94.2% of participants claimed that they had not been hospitalized in the last 6 months.

### AMR of nasal swab samples

From the 3960 nasal swab samples, 215 (5.4%; 95 CI: 4.8–6.2%) were confirmed to be *S. aureus* by MALDI-TOF MS. All isolates were confirmed by molecular analysis. No ciprofloxacin-susceptible isolates were identified. Of the 215 *S. aureus* isolates confirmed by MALDI-TOF MS, 91.6% (*n* = 197; 95 CI: 87.1–94.6%) exhibited dose-dependent susceptibility to ciprofloxacin. In total, 13.9% (*n* = 30; 95 CI: 9.9–19.2%) of isolates were resistant to linezolid; of these, 96.6% (*n* = 29) were from Syrian refugees. Overall, the rate of MRSA was higher among Syrian refugees (6.7%, 95% CI: 5.8–7.7%) than in the local community (3.2%, 95% CI: 2.4–4.2%; *P* < 0.001) (table 1).

**Table 1:** MRSA positivity in isolates from the local community and Syrian refugees, by province

Province	Local community		Syrian refugees		Total		*P*-value
	*n/N*	% (95% CI)	*n*/*N*	% (95% CI)	*n*/*N*	% (95% CI)	
Gaziantep	28/354	7.9 (5.5–11.2)	35/430	8.1 (5.9–11.1)	63/784	8.0 (6.3–10.2)	0.906
Hatay	5/312	1.6 (0.7–3.7)	14/355	3.9 (2.4–6.5)	19/667	2.8 (1.8–4.4)	0.070
Istanbul	1/37	2.7 (0.5–13.8)	3/113	2.7 (0.9–7.5)	4/150	2.7 (1.0–6.7)	1.000^a^
Izmir	0/3	0.0 (0.0–56.1)	58/841	6.9 (5.4–8.8)	58/844	6.9 (5.4–8.8)	1.000^a^
Mersin	4/516	0.8 (0.3–2.0)	33/542	6.1 (4.4–8.4)	37/1058	3.5 (2.5–4.8)	<0.001
Sanliurfa	8/213	3.8 (1.9–7.2)	26/244	10.7 (7.4–15.2)	34/457	7.4 (5.4–10.2)	0.005
Total	46/1435	3.2 (2.4–4.2)	169/2525	6.7 (5.8–7.7)	215/3960	5.4 (4.8–6.2)	<0.001

CI: confidence interval.

a Fisher’s exact test.

### AMR of stool samples

The presence of VRE and Gram-negative bacteria was investigated in stool samples from 1869 participants. VRE was not isolated from any of the stool samples, whereas 96.7% (*n* = 1869; 95% CI: 95.8–97.4%) of samples contained Gram-negative bacteria. Owing to the low number of samples containing *Acinetobacter* spp. (*n* = 3), *Pseudomonas* spp. (*n* = 2) and *Stenotrophomonas maltophilia* (*n* = 1), these species were not included in the analysis. Of the Gram-negative isolates, 1729 (92.5%; 95% CI: 91.2–93.6%) were *Escherichia coli*, 62 (3.3%; 95% CI: 2.6–4.2%) were *Klebsiella pneumoniae*, 7 were *Enterobacter* spp., 2 were *Klebsiella oxytoca* and 1 was *Citrobacter freundii*. Table 2 shows the results of antibiotic susceptibility testing of *E. coli* isolates from stool samples. *Escherichia coli* isolates from Syrian refugees had higher rates of resistance to cephalosporins (cefotaxime and cefepime), piperacillin–tazobactam and gentamicin (see table 2 for *P* values). Only the rate of ciprofloxacin resistance was higher in the local community than in Syrian refugees (*P* = 0.049). 6.3% (*n* = 109; 95% CI: 5.3–7.5%) of *E. coli* isolates, and 69.3% (*n* = 43; 95% CI: 57.0–79.4) of *K. pneumoniae* isolates were ESBL positive, as were one *K. oxytoca* isolate and two *Enterobacter* spp. isolates. Additionally, 22.6% (*n* = 14; 95% CI: 14.0–34.4%) of *K. pneumoniae* isolates were multi-drug resistant; all of these isolates were ESBL positive. In contrast, only 2.8% (*n* = 48; 95% CI: 2.1–3.7%) of *E. coli* isolates were multi-drug resistant; all of these isolates were ESBL-positive. Overall, the study found ESBL-positivity rates of 17.9% (95% CI: 15.6–20.5%) in Syrian refugees and 14.3% (95% CI: 12.0–16.9%) in the local community (*P* = 0.041). No statistically significant differences in ESBL positivity were found among isolates from different provinces (table 3). Furthermore, no statistically significant relationship was identified between ESBL positivity and antibiotic use or hospitalization in the previous 6 months. Phenotypic analysis identified AmpC in 1.1% (*n* = 19; 95% CI: 1.1–1.7%) of *E. coli* isolates and 12.9% (*n* = 8; 95% CI: 6.7–1.7%) of *K. pneumoniae* isolates; of the AmpC-positive isolates, 62.9% (*n* = 17; 95% CI: 44.2–78.5%) were from Syrian refugees. In all, 95.9% (*n* = 47; 95% CI: 86.3–98.9%) of ESBL-positive *E. coli* isolates and 100% (*n* = 43; 95% CI: 91.8–100.0%) of ESBL-positive *K. pneumoniae* isolates (*n* = 17; 79.0%; 95% CI: 67.4–87.53%) were multi-drug resistant, with 75.6% (*n* = 68; 95% CI: 67.8–83.3%) of these from Syrian refugees (table 4).

**Table 2:** Antibiotic susceptibility of *E. coli* isolates from the local community and Syrian refugees^a^

Antibiotic	Local community (*n*=785)			Syrian refugees (*n*=944)			Total (*n*=1729)			*P*-value^b^
	Susceptible (%)		R (%)	Susceptible (%)		R (%)	Susceptible (%)		R (%)	
	S	I		S	I		S	I		
Cefepime	83.4	4.1	12.5	82.8	1.8	15.4	83.1	2.8	14.1	0.006
Cefotaxime	94.5	1.4	4.1	90.1	2.0	7.8	92.1	1.7	6.1	0.003
Ceftazidime	66.2	16.7	17.1	64.1	15.9	20.0	65.1	16.3	18.7	0.291
Ceftriaxone	83.5	1.9	14.5	82.8	1.0	16.2	83.1	1.4	15.5	0.162
Ciprofloxacin	92.6	1.4	6.0	89.4	7.9	2.6	90.9	2.1	7.1	0.049
Ertapenem	95.8	1.8	2.4	95.7	1.1	3.3	95.7	1.4	2.9	0.163^c^
Gentamicin	96.8	–	3.2	93.4	–	6.6	95.0	–	5.0	0.001
Imipenem	100.0	0.0	0.0	99.7	0.3	0.0	99.8	0.2	0.0	0.256^c^
Meropenem	100.0	0.0	0.0	99.6	0.4	0.0	99.8	0.2	0.0	0.131^c^
Piperacillin–tazobactam	82.2	–	17.8	73.7	–	26.3	77.6	–	22.4	<0.001

I: at increased exposure; R: resistant: S: at standard dose.

a *n *=* *1729.

b The local community vs. Syrian refugees.

c Fisher’s exact test.

**Table 3:** ESBL-positive *E. coli* positivity in isolates from the local community and Syrian refugees, by province

Province	Local community		Syrian refugees		Total		*P-*value
	*n*/*N*	% (95% CI)	*n*/*N*	% (95% CI)	*n*/*N*	% (95% CI)	
Gaziantep	25/197	12.7 (8.7–18.1)	59/302	19.5 (15.5–24.4)	84/499	16.8 (13.8–20.4)	0.046
Hatay	24/194	12.4 (8.5–17.8)	35/232	15.1 (11.1–20.3)	59/426	13.8 (10.9–17.5)	0.419
Istanbul	1/9	16.7 (2.0–43.5)	8/46	17.4 (9.1–30.7)	9/52	17.3 (9.4–29.7)	1.000^a^
Izmir	1/3	33.3 (6.2–79.2)	11/69	15.9 (9.1–26.3)	12/72	16.7 (9.8–27.0)	0.426^a^
Mersin	31/231	13.4 (9.6–18.4)	10/109	9.2 (5.1–16.1)	41/340	12.1 (9.0–16.0)	0.262
Sanliurfa	30/154	19.5 (14.0–26.5)	46/168	24.7 (21.2–34.6)	76/340	22.4 (18.3–27.1)	0.977
Total	112/785	14.3 (12.0–16.9)	169/944	17.9 (15.6–20.5)	281/1729	16.3 (14.6–18.1)	0.041

CI: confidence interval.

a Fisher’s exact test.

**Table 4:** The distribution of variables by MDR in the local community and Syrian refugees

Variable	Category	MDR^a^	Total	MDR % (95% CI)	*P*-value
Participants	Syrian	12	785	1.5 (0.9–2.7)	0.003
	Turkish	37	944	3.9 (2.9–5.4)	
Sex	Female	28	877	3.2 (2.2–4.6)	0.478
	Male	17	660	2.6 (1.6–4.1)	
Age	Mean ± SD	34.3 ± 16.1	31.6 ± 16.6	N/A (N/A)	0.311
Use of antibiotics during the last 6 months	Yes	11	519	2.1 (1.2–3.8)	0.205
	No	33	1011	3.3 (2.3–4.5)	
Hospitalization in the last 6 months	Yes	4	47	8.5 (3.4–20.0)	0.046
	No	41	1485	2.8 (2.0–3.7)	
Province	Gaziantep	18	499	3.6 (2.3–5.6)	0.519
	Hatay	15	426	3.5 (2.1–5.7)	
	Istanbul	1	52	1.9 (0.3–10.1)	
	Izmir	1	72	1.4 (0.2–7.5)	
	Mersin	6	340	1.8 (0.8–3.8)	
	Urfa	8	340	2.4 (1.2–4.6)	
ESBL	Positive	47	281	16.7 (12.8–21.5)	<0.001
	Negative	2	1448	0.1 (0.03–0.5)	

CI: confidence interval; ESBL: extended-spectrum beta-lactamases; MDR: multi-drug resistance; SD: standard deviation.

a MDR: multi-drug resistance is defined as combined resistance to third-generation cephalosporins [cefotaxime or ceftazidime or ceftriaxone, fluoroquinolones (ciprofloxacin) and aminoglycosides (gentamycin)].

## Discussion

Global evidence on AMR in migrants is limited. Linked to the recent influx of migrants to the WHO European Region, the number of studies on this topic is increasing.^10^ Türkiye hosts a large number of refugees, including Syrians and other nationals from the Global South. This is the first study to compare the prevalence of AMR carriage including Syrian refugees and the local community members in Türkiye. This study found MRSA prevalence rates of 6.7% in Syrian refugees and 3.2% in the local community, and ESBL-positivity rates of 17.9% in Syrian refugees and 14.3% in the local community. The data show that although MRSA was the most common type of AMR detected among both Syrian refugees and Turkish residents, it was significantly more prevalent among Syrian refugees, and ESBL positivity was found to be statistically significantly higher among Syrian refugees. We suggest immigrants and refugees should be screened for MDR at their first admission to the country. This study provides important data in terms of the surrogacy of MDR in Syrian refugees. The microorganisms investigated in the study are important causes of both community-acquired and hospital-acquired infections. As these bacteria can often cause endogenous infections, identifying their carriers is especially important for determining empirical treatment at the local level, developing policies on eliminating AMR, reducing mortality and morbidity due to AMR and supporting antimicrobial governance. Integrating local and national AMR data with the global AMR database is important for global AMR management.^11^

Studies among Syrian refugees in the WHO European Region have generally used samples from hospitalized patients. A study of migrants in Finland between 2010 and 2017 reported an MRSA carriage rate of 20.9% and an ESBL-positivity rate of 48.8%.^12^ In our study, these rates were higher. However, the AMR rate we found was lower than an Iraqi study, which reported an MRSA carriage rate of 13.8% in Syrian refugees, with a similar rate in Iraqi citizens (15.4%).^13^ We suggest that Türkiye’s location between Africa, Asia and Europe influences its AMR pattern: AMR rates in Türkiye are generally lower than in Asian countries but higher than in European countries.^12–15^

In a study from the Netherlands, the MRSA carriage rate was 9.7% among migrants. In Gram-negative bacteria isolated from migrants, the ESBL-positivity rate was 20%, the quinolone resistance rate was 4.4% and the carbapenem resistance rate was 0.4%.^16^ Since the present study, instead of infectious agents, investigated bacteria in the nasal and gut flora only, the rate of MRSA carriage was lower than in the other studies. High rates of ESBL positivity were identified in both the local community and Syrian refugees. This finding may relate to the overuse of cephalosporin group antibiotics in Türkiye.^17^ AMR carriage among Syrian refugees was unknown when they arrived in Türkiye as there is no baseline data available. Further comparative population-based data are needed to analyze AMR rates in these population groups.

Evidence-based data on pre-conflict AMR rates in Syria are very limited. However, the limited available data suggest that the overuse of antibiotics and poor antibiotic stewardship were significant problems.^18^ The fact that the studies obtained were limited to the cities of Damascus and Aleppo does not allow us to make an evaluation of the country in general. Despite the limited data available, there is evidence that resistance, especially carbapenem resistance, was quite high.^18^

A 2018 meta-analysis of data on refugees and migrants in Europe suggested that AMR patterns in this population may be affected by factors in transit and destination countries. Although there is evidence transmission may occur between migrants, there is no evidence of transfer from refugees and migrants to the host population.^2^

The study identified a high number of multi-drug-resistant isolates in both the local community and Syrian refugees. The high carriage frequency for these isolates within the community may be an important causative factor in a future AMR pandemic. Five of the isolates were also resistant to carbapenems; carbapenemase enzymes were detected in three of these: The New Delhi metallo-β-lactamase (NDM) in two and OXA-48 in one. Türkiye is endemic for OXA-48.^19^ The fact that the OXA-48 carrier had been hospitalized in the previous 6 months suggests that they had acquired the isolate during hospitalization. This finding is consistent with a previous report suggesting that the Middle East may be a reservoir of NDM-producing isolates,^20^ but more data are needed to explain the origin of these NDM-producing isolates. Overall, the rate of carbapenemase-producing isolates in the present study was similar to those of other studies.^13–15^ Further studies, especially on AmpC-positive isolates, carbapenem-resistant isolates and MRSA isolates for high-risk clones, will improve the value of this study. However, due to budgetary limitations and COVID-19 restrictions, further studies could not be carried out within the scope of this study.

The present study underlines the importance of analyzing data from all population sub-groups to better understand their AMR patterns and determine how best to prevent and control the spread of resistant organisms across society. The high rate of AMR carriage highlights the need for longitudinal studies, particularly in light of other research on types and rates of resistant bacterial isolates and how this may change over time in people who have lived as refugees for long periods.^21^ Periodic data collection and analysis may reveal clonal relationships between isolates and allow proactive approaches to monitor and prevent AMR. Such studies may also reveal epidemiological differences between migrant and local populations, and even between migrants from different countries. Therefore, the disease prevalence and carriage of multi-drug-resistant isolates in different population groups should be monitored through a surveillance system that supports international data sharing on AMR. Furthermore, clinical approaches for refugees and migrants should not isolate these groups from society and support their longer-term engagement with health services.^22^

In a review paper, various aspects of AMR in Syrian refugees were discussed. The paper highlights the importance of AMR as a public health problem, which continues to spread in many countries as Syrian refugees migrate. It is emphasized that global health problems such as AMR need to be addressed with a ‘one-health’ approach. It is also stated that standard management practices should be adopted in the region for refugees to maintain the sustainability of public health measures.^23^

In the WHO European region, Türkiye is among the countries with the highest use of antibiotics in outpatient settings. Antibiotic usage also changes seasonally.^11^ In our study, about 63% of the participants claimed that they had used antibiotics in the last 6 months. While it is possible to hypothesize that there is an association between antibiotic use and resistance, we need more evidence for this. However, limiting the use of antibiotics may be one of the strategies to reduce resistance.

The most important limitation we faced was the interruption of data collection due to the COVID-19 pandemic and a major earthquake in the country. Since reliable data on the arrival time of Syrian refugees in Türkiye were unavailable, the study could not consider the time spent in the host country. Participants were limited to people who visited FHCs and RHCs. Another important limitation is the time of the data collection. All Syrian refugees have been in the Turkish community since 2011, first in the refugee camps but after 2014 mostly living outside the camps. So, the year 2020 is considerably late to evaluate and compare the AMR carriage status.

Another limitation was that data were collected from different centers in six provinces. The standardization of study protocols, including collecting epidemiological data, clinical samples and transferring samples, was therefore a challenge. To ensure standardization, structured training was given to healthcare personnel and data collectors in Turkish and Arabic. Another limitation was the time constraint on using enrichment broths for MDR bacteria. During the study period, the study team was also required to work in the COVID-19 pandemic laboratory.

## Conclusion

The civil war in Syria and the consequent population dynamics have significantly contributed to the ongoing public health challenges in the WHO European Region. AMR is an important public health problem in the Region, which originates from healthcare disparities, poor living conditions, inadequate hygiene and sanitation, lack of or outdated treatment guidelines and overuse of antimicrobials. The civil war and, consequently, the deterioration in living conditions and public health infrastructure were likely to have exacerbated this problem.

This is the first large-scale study on the carriage of AMR in Türkiye including both refugees and the local populations; as such, it provides an important insight essential for the effective management of AMR in both Türkiye and the region. Our data can inform screening studies and public health policy decision-making, as well as facilitate accessible high-quality healthcare for both migrant and local populations to support the timely detection and treatment of AMR. Phylogenetic analysis of bacterial isolates obtained over time may provide further data on AMR for both Türkiye and countries on migratory routes.

## Supplementary Material

ckad119_Supplementary_Data

## Data Availability

Data can be shared upon request.
