# Supplementary material for: Community-acquired antimicrobial resistance among Syrian refugees and the local population in Türkiye
Source: Eur J Public Health. 2023 Jul 19;33(5):809–14. doi: 10.1093/eurpub/ckad119 (PMC10567246; doi:10.1093/eurpub/ckad119)
Supplement: ckad119_Supplementary_Data [file ckad119_supplementary_data.zip › ckad119_Supplementary_Data/ejph-2022-05-om-0266-File002.pdf]

Supplementary Figure 1. Workflow for the study

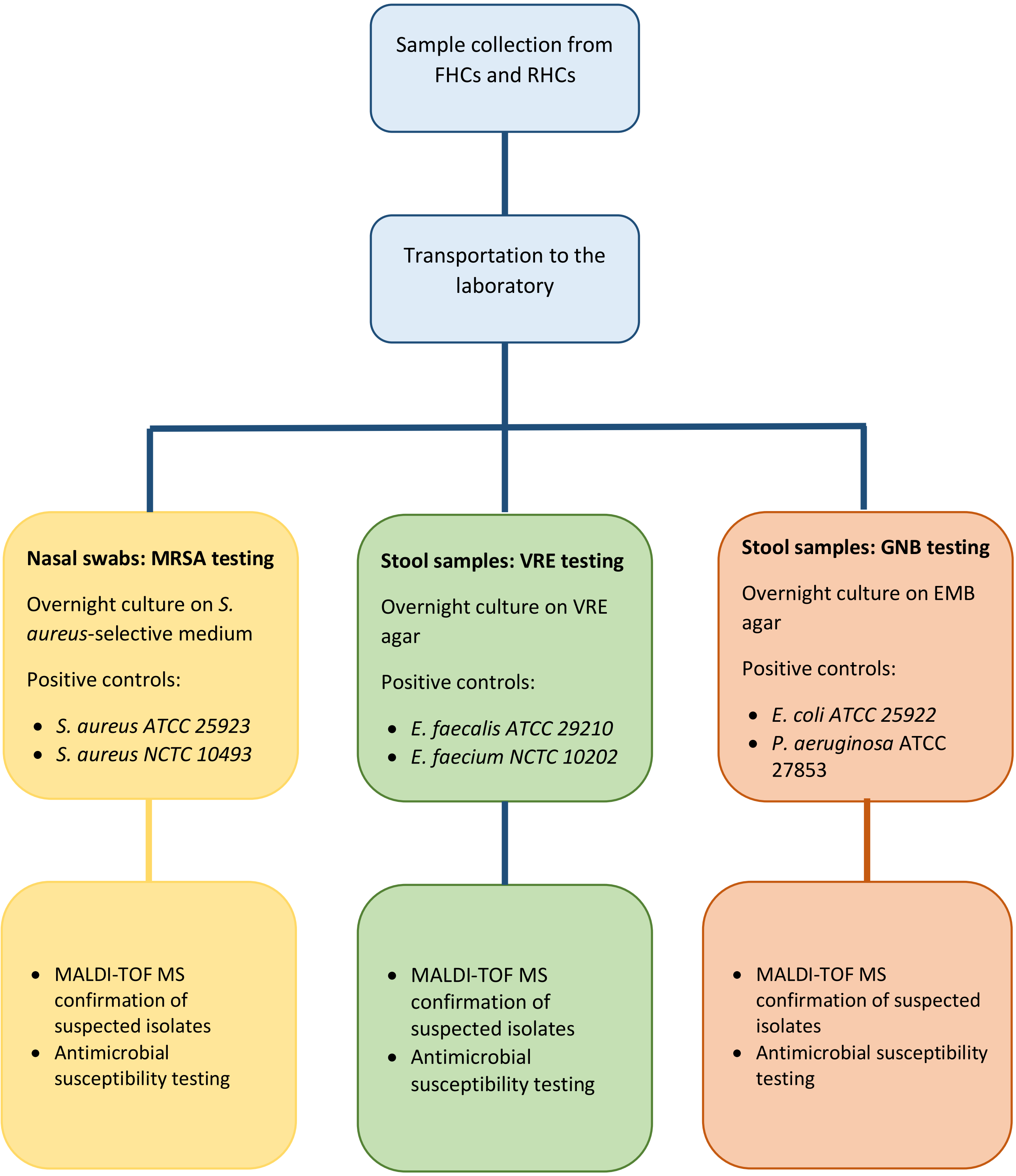

FHCs: Family Health Centers, RHCs: Refugee Health Centers, MRSA: Methicillin Resistant *Staphylococcus aureus*, VRE: Vancomycin Resistant Enterococcus, GNB: Gram-negative bacilli.
