# Supplementary material for: Community-acquired antimicrobial resistance among Syrian refugees and the local population in Türkiye
Source: Eur J Public Health. 2023 Jul 19;33(5):809–14. doi: 10.1093/eurpub/ckad119 (PMC10567246; doi:10.1093/eurpub/ckad119)
Supplement: ckad119_Supplementary_Data [file ckad119_supplementary_data.zip › ckad119_Supplementary_Data/ejph-2022-05-om-0266-File003.pdf]

Supplementary Table 1 Number of clinical samples from the local community and Syrian refugees, by province

Province	Local community		Syrian refugees		Total	
	Nasal swabs (*n*)	Stool samples (*n*)	Nasal swabs (*n*)	Stool samples (*n*)	Nasal swabs (*n*)	Stool samples (*n*)
**Gaziantep**	354	207	430	319	784	526
**Hatay**	312	206	355	269	667	475
**Istanbul**	37	6	113	50	150	56
**Izmir**	3	3	841	75	844	78
**Mersin**	516	247	542	121	1 058	368
**Sanliurfa**	213	171	244	195	457	366
**Total**	1435	840	2525	1029	3960	1869
